# Regulation of K^+^-Dependent Na^+^/Ca^2+^-Exchangers (NCKX)

**DOI:** 10.3390/ijms24010598

**Published:** 2022-12-29

**Authors:** Maryam Al-Khannaq, Jonathan Lytton

**Affiliations:** Department of Biochemistry & Molecular Biology, Libin Cardiovascular Institute and Hotchkiss Brain Institute, Cumming School of Medicine, University of Calgary, Calgary, AB T2N 4Z6, Canada

**Keywords:** potassium-dependent sodium-calcium exchange, Ca^2+^ transport, subcellular location, trafficking, expression, genetics, post-translational modification, allosteric regulation, phosphorylation, palmitoylation, interaction partners

## Abstract

Potassium-dependent sodium-calcium exchangers (NCKX) have emerged as key determinants of calcium (Ca^2+^) signaling and homeostasis, especially in environments where ion concentrations undergo large changes, such as excitatory cells and transport epithelia. The regulation of NCKX transporters enables them to respond to the changing cellular environment thereby helping to shape the extent and kinetics of Ca^2+^ signals. This review examines the current knowledge of the different ways in which NCKX activity can be modulated. These include (i) cellular and dynamic subcellular location (ii); changes in protein expression mediated at the gene, transcript, or protein level (iii); genetic changes resulting in altered protein structure or expression (iv); regulation via changes in substrate concentration (v); and post-translational modification, partner protein interactions, and allosteric regulation. Detailed mechanistic understanding of NCKX regulation is an emerging area of research with the potential to provide important new insights into transporter function, the control of Ca^2+^ signals, and possible interventions for dysregulated Ca^2+^ homeostasis.

## 1. Introduction

Transient and spatially discrete changes in intracellular ionized calcium (Ca^2+^) concentration are universally used to convey a host of different biological signals involved in cellular processes that range from fertilization, cell proliferation, muscle contraction, neurotransmission, and hormonal signaling to cell death. Consequently, [Ca^2+^] is tightly regulated by a constellation of different proteins [1]. Differences in the temporal and spatial control of Ca^2+^ signals are interpreted within the context of the individual cellular protein environment to provide the specificity needed for particular physiological outcomes. Achieving this degree of sophistication in Ca^2+^ signaling requires the careful and integrated regulation of pathways that mediate the initiation, sensing, and termination of the Ca^2+^ signal [2,3,4]. Meanwhile, the dysregulation of Ca^2+^ homeostasis leads to destructive consequences and underlies many disease processes, emphasizing the critical role of this cellular signaling pathway [2].

The resting concentration of cytosolic Ca^2+^ is generally in the vicinity of 100 nM. Signal initiation under the control of extracellular environmental chemical, electrical, or mechanical stimuli results from the opening of channel proteins that allow Ca^2+^ to flow into the cytoplasm from the extracellular space or from intracellular stores, such as the endoplasmic reticulum. The rise of Ca^2+^ is then both sensed and buffered by proteins that bind it. Finally, the signal is terminated by transport proteins that pump Ca^2+^ out of the cytoplasm or back into intracellular storage sites [2,3,4].

The proteins that help terminate the Ca^2+^ signal include the mitochondrial uniporter complex [5], Ca^2+^-ATPase pumps of the plasma membrane (PMCA) [6], endoplasmic reticulum (SERCA) [7], secretory pathway (SPCA) [7], and Na^+^/Ca^2+^-exchangers of the K^+^-independent (NCX) [8] and K^+^-dependent (NCKX) families [9,10]. There are five members of the NCKX family (NCKX1-5, products of the *SLC24A1-5* genes) that exists as a clade in the CaCA superfamily of Ca^2+^/cation antiporters [11,12].

As a side-note, the family member originally denoted NCKX6 [13] is now known to be the mitochondrial Na^+^/Ca^2+^-exchanger, also referred to as NCLX and encoded by the gene *SLC8B1* [14], which is part of a separate clade. NCLX is not K^+^ dependent. Although the Na^+^:Ca^2+^ stoichiometry of NCLX is somewhat controversial [14], recent work suggests a likely coupling ratio of 2 Na^+^ ions to 1 Ca^2+^ ion [15]. This distinct stoichiometry poises NCLX to play a critical role in the regulated release of Ca^2+^ from mitochondria, a process heavily controlled by mitochondrial pH, membrane potential, Ca^2+^ levels, and kinase-mediated phosphorylation [14]. As a consequence, NCLX function is essential for mitochondrial health, and its dysregulation or loss of function are associated with serious disease and death [16,17,18,19].

All the NCKX transporters are thought to couple the transport of 4 Na^+^ ions in exchange for 1 Ca^2+^ ion and 1 K^+^ ion [9,10]. Thermodynamically, this stoichiometry enables NCKX proteins to maintain a Ca^2+^ efflux function even in the face of a depleted Na^+^ gradient and reduced membrane potential. Such properties are ideally suited to environments where ion fluxes are high and ion concentrations fluctuate widely, such as found in excitable cells or secretory epithelia.

The members of the NCKX family have been named numerically in the order of their discovery, characterization, and cloning [20,21,22,23,24]. NCKX1 is expressed almost exclusively in the outer segments of the rod photoreceptors, where it is the only Ca^2+^ extrusion pathway [25]. In the dark state, cyclic nucleotide-gated monovalent ion channels (CNGC) are open, and an exchanger with an NCKX-type stoichiometry is required to maintain Ca^2+^ homeostasis. Upon illumination, the change in [Ca^2+^] that is mediated and temporally shaped by NCKX1 activity is a critical factor for the kinetics and termination of the photo-response and for adaptation to changing light levels [26]. Similarly, important functions in signal termination and adaptation in the cone photoreceptors are determined by the combined actions of NCKX2 and NCKX4 [26] and in olfactory sensory neurons by NCKX4 [27]. 

NCKX2 is widely expressed in neurons throughout the brain [21] in addition to cone photoreceptors [28] and is thought to be a key component of neuronal Ca^2+^ efflux when intracellular Ca^2+^ levels rise above baseline [29]. The gene knockout of Nckx2 in mice leads to deficits in experience-dependent motor learning and memory [30], probably due to dysregulated dendritic Ca^2+^ handing and kinase activation, consequently resulting in the loss of synaptic plasticity in the hippocampus where the protein is abundantly expressed [31,32].

NCKX3 is much more broadly distributed than either NCKX1 or NCKX2, but it is also abundant in the brain [22]. The protein also appears to have a role in Ca^2+^ handing in uterine endometrium and in renal epithelial cells [33,34]. The knockout of Nckx3 in mice leads to modest changes in bone density, susceptibility to colitis, as well as deficits in motor function and social behaviour [35,36,37].

NCKX4 has broad tissue expression and abundance in brain, similar to NCKX3 [23]. Aside from its role in visual and olfactory sensory neurons noted above, NCKX4 has also been shown to impact dental enamel formation [38] and neuronal satiety pathways [39]. 

NCKX5 was first characterized in zebrafish as the gene responsible for the pigmentation mutant, *golden* [24], and subsequently shown to play an important role in melanosome maturation in humans [40].

Thus, it is clear that NCKX family members contribute to diverse physiological responses in different cells and tissues, all linked through a role in shaping the spatial and temporal nature of cellular Ca^2+^ signals. The requirement for sensitive and complex control over Ca^2+^ signals, to ensure fidelity of the downstream biological processes they control, implies that the activity of the NCKX exchangers is carefully regulated. Evidence for regulatory processes is revealed in rod photoreceptors where Na^+^-induced NCKX1-mediated Ca^2+^ extrusion ceases after a few seconds, well before reaching equilibrium ion concentrations [41], and in brain neurons, where purinergic stimulation activates NCKX-driven Ca^2+^ flux [42]. Nevertheless, this is an under-explored area of investigation.

**Figure 1:**
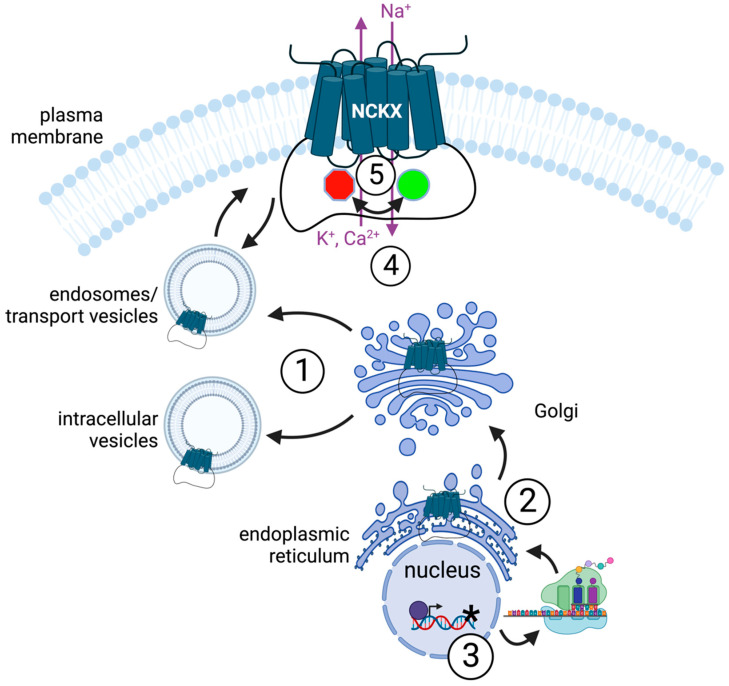
A schematic cartoon illustrating various means by which NCKX can be regulated. The numbers refer to each general mode of regulation covered in a separate section of the review. 1, *cellular location*. NCKX isoforms may reside in different cellular sites due to either biosynthetic trafficking to different membrane compartments—post-Golgi intracellular vesicles *versus* the plasma membrane via transport vesicles—or regulated redistribution between membrane compartments—such as endosomes and the plasma membrane. 2, *protein expression*. Regulation of expression can take place at the transcriptional, mRNA stability, translational, protein stability and/or trafficking levels. The transcribed gene is shown translated into NCKX proteins which are then incorporated into the endoplasmic reticulum and subsequently trafficked through the Golgi to either an intracellular location or via transport vesicles to the plasma membrane. 3, *genetic changes*. Mutations in or associated with the NCKX (*SLC24*) genes (*asterisk*) or alternative splicing of transcripts can influence protein structure and activity, and/or expression levels. 4, *substrate concentration*. The ion substrates for NCKX are shown. Changes in their concentration can alter the rate—and potentially the direction—of transport by changing occupancy of the transport sites. Ions can also interact at a distance from the transport sites to regulate activity allosterically. 5, *post-translational modification and allosteric regulation*. The activity of the NCKX protein in the membrane can be influenced directly by various modifications and interactions, here denoted by the *red* “stop” to *green* “go” transition. This mode of regulation is also illustrated in greater detail in Figure 2, below.

This review will focus on current knowledge of the different ways in which the function of NCKX proteins can be modulated, including changes in cellular location, expression levels, genetic changes, substrate concentration, and post-translational modifications. These different modes of regulation are illustrated schematically in Figure 1.

## 2. Cellular Location

Different NCKX genes are expressed, and their protein products reside, in different cells and tissues, thus linking them to different physiological processes such as rod photoreceptor signaling (NCKX1), olfactory sensory neuron signaling (NCKX4), or skin melanocyte maturation (NCKX5). Nevertheless, there is no clear indication that the different NCKX proteins display distinct functional properties tailored to these different cellular environments and processes [9,10]. Perhaps a notable exception to this general fact is subcellular location, which varies among the different NCKX proteins.

Subcellular location is an important determinant of the contribution NCKX proteins make to Ca^2+^ signals and downstream biological consequences. For example, while NCKX1 and NCKX2 are well-documented to reside mainly at the plasma membrane [26,43,44,45,46,47], NCKX5 is present intracellularly, in a trans-Golgi or post-Golgi membrane compartment [40], and associated with mitochondria [48]. In the case of NCKX1 and NCKX2, the exchangers engage in Ca^2+^ efflux from the cell while in the case of NCKX5, the exchanger is involved in Ca^2+^ transport across an organellar membrane—although the mechanisms linking NCKX5 expression and transmembrane Ca^2+^ movement to melanogenesis are not well understood [40,49], but may involve the transfer of Ca^2+^ from mitochondria mediated by NCKX5 [48]. The subcellular location of expression for NCKX2 and NCKX5 is preserved when these proteins are expressed in heterologous cell systems [21,50]. Employing such a system, it was found that the signals dictating the compartment in which NCKX2 or NCKX5 reside appear to be present in the central cytoplasmic loop of the protein, although no single short segment or simple motif responsible could be identified [50].

The finding that NCKX5 resides in an intracellular membrane compartment (and see below regarding regulated trafficking of NCKX2 and NCKX4 to intracellular vesicles) raises the issue of ion gradients and the direction of Ca^2+^ transport in such compartments. As noted above, NCKX proteins on the plasma membrane can harness the large inward Na^+^ and outward K^+^ gradients, as well as the negative membrane potential, to drive Ca^2+^ efflux. The post-Golgi compartment associated with melanosome biogenesis is considered part of the lysosome-related organellar and endosomal network. Here, the nature of the existing ion gradients is less clear [24,48,51,52]. There is good evidence for Ca^2+^ accumulation within the vesicles of this network; [K^+^] is thought to be close to the level found in the cytosol; the membrane potential appears to be low; and the pH is known to be low. The pH gradient is maintained by the V-ATPase and can increase luminal [Na^+^] via Na^+^/H^+^-exchangers. This small Na^+^ gradient may be sufficient to allow Ca^2+^ accumulation because of the 4Na^+^:1Ca^2+^ transport stoichiometry of NCKX proteins [24].

While NCKX2 expressed in HEK293 cells has a relatively uniform distribution on the cell surface [21,50], in mature differentiated neurons, the protein is located primarily at cellular processes distant from the cell body, including both small distal dendrites and axon terminals. Interestingly, the balance of distribution between these two locations is different in different brain regions [31]. It has been proposed that NCKX2 is initially targeted to the soma and dendritic tree and subsequently endocytosed and trafficked to the axon terminal. This redistribution requires the motor kinesin KIF21A, which binds to the cytosolic loop of NCKX2 via WD40-repeat domains [46]. In keeping with this model, NCKX2 is also observed within dendritic structures, presumably on transport vesicles, particularly in brain regions where distribution to the axon terminals is high [31]. The somatodendritic endocytosis of NCKX2 required for axonal targeting depends on an interaction between a tyrosine residue in the protein’s large cytosolic loop and the clathrin adaptor protein, AP-2 [53]. This tyrosine, present in a protein segment subject to alternative splicing [21], is a target for Src-family kinase phosphorylation, a process regulated by Ca^2+^ signaling as well as other pathways including the protein kinase PYK2 [53]. Thus, the cellular destination of NCKX2 can be influenced both by the nature of the alternatively spliced isoform expressed in a particular cell, and by dynamic regulation of the endocytic and trafficking processes that move NCKX2 from the somatodendritic membrane to the axon terminal. These processes may differ in different neurons, thereby resulting in NCKX2 distribution skewing toward distal dendrites in some neurons, but axon terminals in others. Consequently, NCKX proteins will influence Ca^2+^ signals underlying synaptic integration in one case (dendrites) [30,31] or synaptic vesicle release (axon terminals) [44,54] in the other.

NCKX4 has been demonstrated to play an important role in Ca^2+^-dependent dental enamel deposition and is thought to be essential for apical Ca^2+^ secretion from dental ameloblasts [38,55]. Consistent with this role, NCKX4 is found concentrated at or near the apical membrane of maturation-stage ameloblasts. Ameloblasts of this stage cycle between two states, one with a ruffled apical membrane, and one with a smooth apical surface. NCKX4 is found predominantly near the apical surface of ruffled ameloblasts but within the cytoplasm of smooth cells [55]. Interestingly, the enamel-deficient state induced by high dietary fluoride content causes a redistribution of NCKX4 from the apical end of the maturation-stage ameloblasts toward the cytoplasm [56]. In addition, defects in the protein WDR72, which is found on intracellular vesicles in maturation-stage ameloblasts, interfere with mineralization and enamel maturation, and result in the redistribution of NCKX4 from the apical membrane to intracellular vesicles [57]. These observations suggest that the final destination of NCKX4 may be a dynamically regulated process that depends upon the physiological state of the cells where it is expressed. Intriguingly, like NCKX2, NCKX4 possesses a short tyrosine-containing segment in the large cytosolic loop that is subject to alternative splicing. However, unlike NCKX2, there are no studies examining possible mechanisms regulating redistribution of NCKX4 between different membrane compartments. The subcellular location of NCKX4 and its potential dynamic regulation in dental ameloblasts, brain neurons, and olfactory and visual sensory neurons is an important area for future investigation.

A further consequence of subcellular location on the function of NCKX proteins is the possible interaction with other membrane proteins targeted to a similar domain. For example, the rod NCKX1 protein has been demonstrated to exist in situ in a complex with the CNGC subunit, CNGA1 [58]. There is also evidence that in cones, NCKX2 may interact with CNGA3 [59]. The proximity of CNGCs with NCKXs might create a local microdomain of ions that could shape Ca^2+^ signals and thus modulate the phototransduction response [26]. These concepts are explored in more detail in subsequent sections of this review below.

## 3. Protein Expression

There is strong evidence for an important quantitative role of NCKX proteins, particularly NCKX2, in neuronal Ca^2+^ extrusion [30,44,47]. In addition, gene knockout studies in mice reveal specific physiological consequences of the loss of expression of NCKX1 [60], NCKX2 [30,32], NCKX3 [35,36,37], and NCKX4 [27,38,39] proteins. Longer-term modulation of NCKX protein expression would therefore be expected to influence Ca^2+^ homeostasis, and the possibility exists for important compensatory changes in NCKX protein expression linked to physiological or pathological changes in cellular Ca^2+^ handling.

Dysregulated Ca^2+^ homeostasis is a key factor underlying serious degenerative brain pathology, particularly in stroke. Experiments in rats and mice using ischemia as an experimental model for stroke reveal both a loss of NCKX2 expression, which may precede and exacerbate subsequent Ca^2+^-induced neuronal degeneration, as well as a neuroprotective effect of NCKX2 expression in the face of ischemia [61]. Intriguingly, recent studies have revealed that ischemic preconditioning, a mild stress that induces resilience to subsequent ischemic events, is associated with upregulation of NCKX2 expression [62]. The importance of increased NCKX2 expression in the ischemic state may be due to the NCKX2 transport stoichiometry (4Na^+^/1Ca^2+^+1K^+^), which allows neuroprotective Ca^2+^ extrusion to be maintained under the reduced ion gradients present in the early ischemic state, although possibly not during severe ischemia and in the extreme case of excitotoxic stress [45,63].

These data suggest a homeostatic loop, whereby physiologically modest sustained increases in cellular [Ca^2+^] lead to an increase in NCKX2 expression. Disruption of homeostasis under the extreme conditions of ischemia, however, breaks the loop, resulting in the collapse of NCKX2 expression. The link between Ca^2+^ signaling and gene expression is well studied, particularly in the context of synaptic plasticity [64]. Nevertheless, the detailed mechanisms that link [Ca^2+^] changes to altered NCKX2 expression remain to be determined.

Several animal models of neuronal development show changes in NCKX2 expression that correlate with the differentiation and maturation of neurons and their synaptic connectivity [65,66]. Additionally, developmental changes in Ca^2+^ handling at synapses have been linked, in part, to changes in NCKX activity [67]. Some drugs and environmental factors have also been linked via altered NCKX2 expression to deleterious changes in neuronal development [68,69]. These changes are consistent with an important role for NCKX2 in maintaining Ca^2+^ flux and homeostasis in healthy neurons during the process of maturation. Studies on bone-derived mouse dendritic cells have also revealed several different pathways regulating Ca^2+^ signaling that result in a change in the expression of NCKX1 and Na^+^/Ca^2+^-exchange activity [70,71].

Alterations in NCKX gene expression in the face of pathological events have also been noted in megakaryocytes in response to TGF-β treatment (reflective of inflammation) [72], in pancreatic islet β-cells in type-2 diabetes [73], in spinal cord neurons signaling neuropathic pain [74], and in a model of cell proliferation [75]. The mechanisms that lead from the initial event or signal to the change in NCKX2 expression have not yet been examined in detail but appear to vary between different cells and tissues. In some cases, such as ischemic preconditioning and inflammation, kinase cascades have been implicated [62,72]. An emerging theme, that might connect the diverse events regulating NCKX2 expression such as neuropathic pain, proliferation, brain ischemia, and retinopathies is the induction or suppression of different microRNA species [74,75,76,77]. This mode of regulation could enable coordinated changes in the expression of a network of related genes. There is also evidence for microRNA-mediated regulation of NCKX4 expression in cerebral infarction and in vascular smooth muscle cells [78,79]. A large increase in NCKX2 phospho-peptides present in serum has also been found in patients with Alzheimer’s disease compared to the control [80]. It is unclear, however, whether this reflects an increase in the release from damaged cells or a change in NCKX2 expression and/or modification.

NCKX3 expression in uterine smooth muscle has been shown to respond during the estrus cycle to changing concentrations of estrogen and progesterone [81]. An association between NCKX3 expression and the arterial disease fibromuscular dysplasia [82] and gynecological cancer [83] have also been reported. Such expression changes may support Ca^2+^ homeostasis in response to changing smooth muscle contractile states under these conditions.

The changes in NCKX expression reported in the literature could come about by changes in transcription, mRNA stability, translation, or protein stability. Aside from the studies (above) implicating miRNA in regulating transcripts, little is known about which steps are involved in regulating NCKX expression. Further studies detailing mechanisms that lead to long-term changes in NCKX protein expression, combined with a deeper understanding of how this impacts physiology and pathophysiology, may reveal interesting new targets for therapeutic intervention in disease states. This seems particularly true for NCKX2 in the context of brain ischemia.

## 4. Genetic Change

Genetic variation has the potential to impact the function of the encoded NCKX exchangers. In this section, we discuss alterations at the genomic DNA and transcriptional levels via polymorphisms/mutations and mRNA processing by alternative splicing. While splicing can be a regulated process, mutation is not “regulation” in its common usage because the subsequent changes in function or expression are neither temporally controlled nor reversible. Mutations are nonetheless important events that influence the physiological processes associated with NCKX activity.

The most obviously identifiable genetic variation is a monogenetic Mendelian link with a disease or a well-defined phenotype. Several NCKX genes have now been clearly linked to human disease. Mutations in *SLC24A1* (NCKX1) cause one of several forms of autosomal-recessive congenital stationary night blindness (CSNB) [84,85]. In the form of CSNB caused by the loss of NCKX1 function, affected individuals have a loss of vision in dim light, good vision in bright light, and no retinal degeneration with age. Thus, the loss of NCKX1 expression in rod photoreceptor cells results in defective phototransduction signaling, although the rod cells remain intact. A mouse Nckx1 knockout model largely recapitulates the human disease and suggests that compensation via other transport pathways allows control of Ca^2+^ homeostasis sufficient to be compatible with cell viability but not with efficient phototransduction [60].

While many polymorphisms have been identified in *SLC24A* (NCKX2), which is expressed in cone photoreceptors, none have been linked to retinal disease or visual defect [86]. Nor have any mutations in *SLC24A4* (NCKX4) been found that cause visual disease. This may be due to the dual involvement of NCKX2 and NCKX4 in cone Ca^2+^ homeostasis and phototransduction [87]. Alternatively, compensatory changes in the expression of other Ca^2+^ handling proteins and transporters may support cone photoreceptor function when NCKX2 or NCKX4 are inactivated, in a manner somewhat like the early adaptation to NCKX1 inactivation, noted above. In this regard, PMCA2 expression has been shown to modulate visual signaling in the retina [88]. Thus, the up-regulation of specific PMCA isoforms may help protect cones from the loss of NCKX2 or NCKX4 function.

No disease-causing mutations have been identified in *SLC24A3* (NCKX3) either.

Although apparently without effect on either vision or smell, several rare mutations in *SLC24A4* (NCKX4) have been identified that cause a form of amelogenesis imperfecta, a disease in which tooth enamel formation and mineralization is impaired [38,89,90,91,92,93]. It might be expected that the mineralization defect that caused the loss of NCKX4 is due to a reduction in apical Ca^2+^ secretion capacity. However, because there are other Ca^2+^ transporters (notably NCX1, NCX3, and PMCA4) present that can extrude Ca^2+^ across the apical membrane [94], it is also possible that the loss of NCKX4 impairs enamel formation by preventing the effective hydrolysis and removal of enamel matrix proteins that are essential for proper mineralization. However, the mechanism by which NCKX4 might modulate this process remains unclear.

When NCKX5 was first characterized, a very common polymorphism in the *SLC25A5* gene was identified that displayed remarkably different allele frequencies in human populations with different levels of pigmentation [24]. The polymorphism changed an ancestral Alanine residue at position 111 to Threonine, which was shown to significantly reduce Ca^2+^ transport activity [40]. Subsequently, mutations in NCKX5 that lead to a form of oculocutaneous albinism were uncovered [95,96,97,98,99].

While the effect of Mendelian genetic mutations on NCKX activity can be dramatic, these instances are very rare. Instead, the association of polymorphisms in NCKX genes with more complex phenotypes and disorders is much more common. Arguably, such changes are more informative with respect to regulation of NCKX activity underlying physiology and pathophysiology.

Whole exome and whole genome sequencing as well as genome-wide association studies of single nucleotide polymorphisms have revealed strong associations between various NCKX genes and human conditions. From these types of analyses, variants in *SLC24A2* (NCKX2) were identified that are associated with pancreatic ductal adenocarcinoma [100] and, surprisingly, skin colour variation in a Chinese population [101]. Variants in *SLC24A3* (NCKX3) have been associated with hypertension [102] and other common cardiovascular diseases [82] as well as migraine [103] and Alzheimer’s disease [104].

Several studies have shown associations between *SLC24A4* (NCKX4) variants and variations in eye, hair, and skin colouration [105,106], which is in keeping with data supporting a role for NCKX4 in Ca^2+^ homeostasis in pigmented melanoma cell lines [107]. However, like NCKX5, the mechanism underlying a possible role for NCKX4 in pigmentation is not clear. An association between *SLC24A4* variants and hypertension has also been suggested [108]. Intriguingly, a plethora of studies have shown a significant association of *SLC24A4* variants with Alzheimer’s disease [109,110,111,112,113,114,115,116], suggesting an important role for NCKX4 in neuronal development and maturation. It is possible that alterations in NCKX4 function in cerebral vascular smooth muscle might result in blood vessel pathology, thus connecting different brain-associated conditions. Such an underlying role for NCKX4 in the vasculature would be in keeping with the data supporting regulation of NCKX4 in the vascular smooth muscle, as noted above [78,79].

All the *SLC24* family genes are subject to alternative splicing as evidenced by cloning studies and RNASeq expression data available in the databases [21,22,23,117]. Splicing impacts both the 5′-untranslated region of NCKX transcripts, as well as regions encoding segments of the central cytoplasmic loop. There is evidence for a particularly complex pattern of possible alternatively spliced products for rat NCKX1 [117] and mouse NCKX2. As noted above, the segments of the protein subject to variation due to alternative splicing contain residues and motifs potentially involved in both phosphorylation and membrane retrieval. However, no experiments have addressed the functional consequences of different alternatively spliced NCKX protein products. Nevertheless, the implications for regulated processes involving different spliced products, as discussed above, suggest this might be an interesting topic for further study.

## 5. Substrate Concentration

Transport protein activity depends upon the concentration of the substrates and their binding affinities at the transport sites. In the case of the NCKX proteins under normal physiological conditions, where the inward movement of Na^+^ ions is in exchange for the outward movement of Ca^2+^ and K^+^ ions, both the outward facing Na^+^ site and the inward facing K^+^ site are effectively saturated, but the inwardly facing Ca^2+^ site with low µM affinity is not [118,119,120,121]. This characterization of apparent affinities for the ion binding sites suggests that the availability of cytosolic Ca^2+^ is a key determinant of transport activity. The suggestion that [Ca^2+^] is a limiting substrate for NCKX activity is borne out by detailed kinetic analysis of different Ca^2+^ transport pathways in neuronal nerve terminals [29]. Here, PMCA is the predominant pathway at basal sub-μM [Ca^2+^] levels, while NCKX activity predominates over the low µM levels found during signaling events, with mitochondrial accumulation playing an increasingly important role as [Ca^2+^] rises even further to deleterious and possibly pathological levels.

An additional kinetic consideration for NCKX activity is the release of transported K^+^ from its extracellular binding site. The affinity for K^+^ here is in the low mM range (except lower for NCKX2 [119,120]), suggesting that Ca^2+^ extrusion activity might be slowed during periods of tetanic nerve activity when extracellular [K^+^] is thought to rise. It is interesting to note that studies on mature neurons do not support the role of NCKXs in Ca^2+^ transport in the soma region [122], which is generally in keeping with the known distribution of NCKXs at peripheral neuronal processes (see above). Neuronal processes are relatively small and are the principal site of changing ionic flux associated with signaling. Consequently, ion concentrations at these sites can vary much more widely than in the larger volume of the cell body.

NCKX proteins, because of their transport stoichiometry linking the movement of 4 Na^+^ ions in one direction to 1 Ca^2+^ ion and 1 K^+^ ion in the other, are thermodynamically poised to transport Ca^2+^ ions out of the cytoplasm under a wide range of ion concentrations and membrane potentials. Nevertheless, under pathological conditions associated with severe ischemia and excitotoxic states, the extent of the alterations in ion concentration can cause the reversal of NCKX transporters, leading to the build-up of toxic levels of Ca^2+^ [45,63].

Substrates can also be allosteric regulators of transporters, as well described for the cardiac NCX1 [123]. In NCX1, Ca^2+^ binding to two well-characterized domains in the cytosolic loop far away from the transport site activates exchange. Meanwhile, Na^+^ acts as an allosteric inhibitor, apparently involving the intracellular ion transport sites themselves, although the molecular mechanism is not well understood [123]. No equivalent binding sites or allosteric mechanisms have been reported for the NCKX proteins [10]. However, two different potential ion-dependent regulatory mechanisms have been described. First, Na^+^-initiated Ca^2+^ flux measurements in bovine rod outer segment preparations demonstrate a time-dependent rapid inhibition of activity that prevents NCKX1 from lowering Ca^2+^ below a threshold value of about 100 nM [41]. Second, a time- and Na^+^-dependent inhibition has been seen when measuring NCKX2 activity in the HEK293 cell expression system using a reverse-mode assay [124]. Although interaction at the intracellular ion transport sites seems to be required, the time-course and character of this process is quite different from the Na^+^-dependent inhibition of NCX1. It is unclear if the time-dependent inhibition of NCKX2 measured in HEK293 cells is related to the Na^+^- and time-dependent inhibition observed for NCKX1 in rod outer segments.

## 6. Post-Translation Modification and Allosteric Regulation

**Figure 2:**
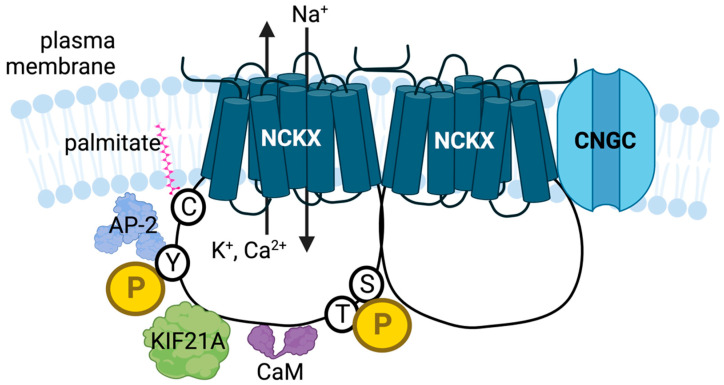
The different known NCKX post-translational modifications and allosteric regulation steps are illustrated. *Protein complex formation*. The NCKX protein is shown in the membrane as a homodimer. In photoreceptors, the NCKX dimer is known to interact with the CNGC subunits, CNGA1 and CNGA3. Interactions with CaM, KIF21A and AP-2 are also illustrated. The latter two proteins likely also influence NCKX distribution between different membrane compartments. *Phosphorylation*. NCKX is modulated by phosphorylation (P) on either tyrosine (Y), serine (S) or threonine (T) residues. SFK-mediated phosphorylation of a Y residue is thought to interfere with the AP-2 interaction required for retrieval of NCKX2 from the plasma membrane into endosomes. CaM is thought to interact with phosphorylation mediated by PKC and/or CaMKII to provide complex temporal control over NCKX4 activity. *Palmitoylation*. Modification of a cysteine (C) residue with palmitate is thought to modulate NCKX4. For simplicity, modifications are only illustrated on one subunit of the NCKX dimer.

Perhaps the most common form of protein regulation is via allostery, most often in the form of post-translational modifications that influence protein conformation and, hence, activity. In this section of the review, we will discuss the currently known processes by which NCKX proteins are allosterically regulated. Figure 2 illustrates these different events.

### 6.1. Protein Complex Formation

Cross-linking and hydrodynamic studies have identified NCKX1 homodimers within the plasma membrane of bovine rod photoreceptor outer segments [125]. Because NCKX1 comprises only a small fraction of the rod outer segment membrane proteins, the specificity of the disulfide-cross-linking indicates a very tightly associated NCKX1 homodimer. Subsequent studies using recombinant proteins established that a single cysteine residue in the N-terminal extracellular domain is required to observe NCKX1 cross-linking [59]. Importantly, the multimeric nature of the NCKX1 complex does not appear to require disulfide cross-linking, but the complex predisposes NCKX1 to this reaction, presumably by bringing the cysteine residues on neighbouring subunits close together [59,125]. As noted above, the NCKX1 homodimer has also been shown to interact with the light activated CNGC of rod outer segments via the CNGA1 subunit [58,59]. The NCKX1-CNGA1 interaction seems to influence the orientation of NCKX1 within the homodimeric complex, leading to a greater efficiency of cross-linking [59]. Additional studies suggest that the NCKX1 homodimer in rod outer segments exists in a partially inhibited state that can be relieved by both cross-linking and by partial proteolysis of the membrane [126]. These authors further suggest that when CNGC is active, the CNGA1-NCKX1 interaction reduces the inhibitory effect between protomers of the NCKX1 homodimer, thereby activating the exchanger. Such an arrangement may help modulate Ca^2+^ levels in close vicinity to the channel through NCKX1-mediated extrusion [58,126]. It seems likely that NCKX2 homodimers form a similar complex with the CNGC subunit CNGA3 in cone photoreceptors [59]. Interestingly, it has also been suggested that NCKX2 homodimers may be stabilized by a disulfide bond between cysteine residues in the central cytosolic loop. This interaction appears to inhibit the exchanger’s activity while breaking the disulfide linkage by reduction may release structural constraints and increase NCKX2 transport activity [127]. Whether such a redox mechanism can account for the dynamic regulation of NCKX2 has not been determined.

This specific arrangement of NCKX homodimers within a protein complex with the CNGC in both rod and cone photoreceptor outer segments may be critical for the control of Ca^2+^ levels and the downstream physiological events in those cells. CNGC is the only Ca^2+^ entry pathway and NCKX is the only Ca^2+^ extrusion pathway in photoreceptor outer segments. Hence, by physically and functionally associating Ca^2+^ entry and exit mechanisms, both the kinetics and spatial extent of Ca^2+^ signaling can be controlled [58,59]. Whether similar complexes between NCKX and ion channels exist in other cellular locations remains to be determined. However, the association of NCKX1, NCKX2, and NCKX4 with time-dependent adaptation of both visual and olfactory sensory responses [27,87], suggests a generally important role of the exchangers in limiting the kinetics of Ca^2+^ signals.

The NCKX-NCKX and NCKX-CNGC interactions are not isoform-specific. When co-expressed together in heterologous systems, NCKX1 can also be co-immunoprecipitated with NCKX2. Similarly, co-expression of NCKX1 or 2 with CNGA1 or 3 results in complexes between either NCKX and CGNA isoforms [59]. It seems likely, however, that these promiscuous interactions are not observed in vivo, where the rod-specific NCKX1 and CGNA1 are not expressed in the same cells as the cone-specific NCKX2 and CGNA3 [26]. However, this may not be true for other NCKX gene products, for instance, NCKX2, NCKX3, and NCKX4, which may be co-expressed within the same cells. The existence and functional consequence of such possible heteromeric complexes have not yet been investigated. 

### 6.2. Phosphorylation

The phosphorylation of proteins represents one of the most common forms of post-translational modification linked to changes in activity. Phosphorylation cascades are well known mediators of regulated Ca^2+^ homeostasis in many environments, including muscle contraction and neuronal plasticity [128,129].

#### 6.2.1. NCKX2 Phosphorylation

NCKX2 has been implicated in the integration of synaptic plasticity [30,32], presumably by shaping Ca^2+^ signals. Therefore, Ca^2+^-dependent pathways that regulate this process, such as PKC, are of particular interest. Indeed, the activation of PKC has been shown to stimulate NCKX activity in axon terminals at the calyx of Held [42]. Studies using recombinant NCKX2 expressed in HEK293 cells demonstrated that NCKX2 stimulation was mediated through a temporally complex mechanism that likely involved the phosphorylation of multiple residues on NCKX2 by a novel-type PKC (PKCε) and their subsequent Ca^2+^-dependent dephosphorylation via calcineurin [42]. While the changes in NCKX-mediated Ca^2+^ flux observed following PKC activation are clear, the molecular mechanisms that allow NCKX to be regulated remain unexplored. It is interesting to speculate how covalent modifications and/or subsequent changes in protein interactions within the NCKX2 cytoplasmic loop can lead to changes in activity. A direct interaction between the cytoplasmic loop and the transport domain in the membrane might mediate these effects, perhaps through the relief of a constraint in the conformational changes associated with transport [130]. Another mechanism to influence activity might involve a redistribution of the transporter from a submembrane pool of vesicles.

As discussed earlier, NCKX2 has a polarized surface distribution on neurons that depends upon somatodendritic endocytosis and subsequent vesicular trafficking to axon terminals mediated by the kinesin KIF21A [46]. Evidence has been presented that phosphorylation of a key tyrosine residue on NCKX2 by an Src family kinase (SFK) can modulate the endocytic process, changing the exchanger’s expression on the cell surface [53]. Aside from playing an important role in controlling the subcellular distribution of NCKX2, SFK-mediated phosphorylation could also act to regulate the activity of NCKX2 at the cell surface. Indeed, NCKX2 activity is enhanced in the proximal dendrites of dentate granule cells when SFK is activated [53]. In this context, SFK can be activated by a variety of pathways related to, among other things, cell remodelling, adhesion, proliferation, differentiation, and stress. These processes are all functionally related to and cross-talk with Ca^2+^ signaling [131]. Thus, it is possible that changes in surface residence, mediated via the SFK pathway, may be involved in the changes in NCKX2 activity observed due to activation of PKC [42].

#### 6.2.2. NCKX4 Phosphorylation

Purinergic signaling, mediated by ATP release, is thought to be an important modulatory component of neurotransmitter-dependent Ca^2+^ signaling dynamics [132]. ATP treatment has been shown to stimulate NCKX4 activity through G-protein coupled purinergic P2Y receptors in a process that requires the dual activation of PKC and CaMKII [133]. This study also demonstrated that the mutation of one candidate PKC site on NCKX4, T312, significantly reduced, although it did not eliminate, the extent of stimulation. The activation of NCKX4 by this pathway may help dampen signaling cascades by the increased extrusion of Ca^2+^ from the cell. The precise mechanism that leads from the stimulation of PKC and CaMKII to an increase in NCKX4 activity has not yet been identified. As with regulation of NCKX2, allosteric activation of transport activity or recruitment to the plasma membrane from a sub-membrane vesicle pool are possible mechanisms. Intriguingly, the activation of GluR1 that underlies synaptic plasticity also requires a synergistic activation of PKC and CaMKII [134].

### 6.3. NCKX4 Regulation by Calmodulin

Calmodulin (CaM) is a very common mediator of Ca^2+^-dependent regulation processes, binding to helical amphipathic basic motifs [135,136,137]. A scan of the NCKX sequences with a web-based tool [138] reveals putative CaM binding sites in the central cytosolic loops of all NCKXs. The interaction of CaM with NCKX4 was confirmed with a yeast-2-hybrid screen [139]. In this study, CaM bound to NCKX4 at basal Ca^2+^ levels, appearing to stabilize the protein but inhibit activity. Following a Ca^2+^ signal, rapid activation of NCKX4 activity associated with PKC/CaMKII phosphorylation helps to extrude Ca^2+^ from the cell, hence dampening the signal. Subsequently, a slow Ca^2+^-dependent reorientation of CaM on NCKX4 causes a reversal of the activation [139]. These complex and antagonistic modes of NCKX4 regulation may be important for shaping cellular Ca^2+^ signaling events. Such a model fits with the role of NCKX4 as an important mechanism for time-dependent adaptation to a signal response in the olfactory and visual systems [27,87]. The regulation of the other NCKXs by CaM has not yet been investigated, although there are some intriguing similarities between the complex time-dependency of NCKX4 activation and that observed for PKC-stimulation of NCKX2 (see above).

### 6.4. NCKX4 Regulation by Palmitoylation

The reversible thioester bond between a protein cysteine and palmitic acid (palmitoylation) is a post-translational modification that regulates many membrane proteins [140,141]. Palmitoylation may affect the protein’s trafficking, cellular localization, activity, or association with other proteins. NCX1 palmitoylation has been studied extensively, revealing modification at a single site at the C-terminal end of the large cytoplasmic loop that influences membrane distribution and activity [142]. The palmitoylation of NCKXs has until now remained unexplored despite sequence similarities to the palmitoylation domain of NCX1. Recently, our laboratory has demonstrated the palmitoylation of NCKX4 at multiple cysteines [143]. The details of this process and its effect on NCKX4 function are still under investigation.

### 6.5. Other Possible Interaction Partners

STRING is a database of protein networks that includes both known and predicted interactions [144]. The entries in STRING are obtained by aggregating data from a variety of methods that include extracting information from existing experimental datasets and databases, text mining of the current literature, and computational predictions based on the co-expression data and conservation of interactions with homologs found in other organisms. Examining the STRING entries for the different NCKX proteins reveals interesting interaction networks with both anticipated and unanticipated partners. The known interactions between NCKX1 and CNGA1, NCKX2 and CNGA3, and NCKX2 and KIF21A were correctly identified in the STRING database.

For the unanticipated interactions, further investigation into the underlying data reveals that in most instances the networks represent a connection between proteins whose expression is known to co-localize in the same cells/tissue with the corresponding NCKX protein or is associated with the same physiological process (e.g., visual transduction with NCKX1; pigmentation with NCKX5; general Ca^2+^ homeostasis with several NCKXs). In other instances, the two partners are simply both mentioned in the text of a publication (for example, if both gene products are found to have altered expression in a particular condition). However, none of these predictions are supported by experimental evidence. Nevertheless, some of the connections between NCKXs and proteins linked by physiological or genetic interactions might represent potentially interesting avenues for future investigation.

In this regard, two examples stand out. First, several NCKX entries in the STRING database highlight a strong genetic interaction observed in *D. melanogaster* between the NCKX homolog, *zydeco*, and CaM [145]. This connection anticipates the experimentally determined NCKX4 and CaM interaction [139], although the latter is not captured by STRING. Second, STRING identifies a connection between NCKX4 and WDR72. Both proteins are co-expressed in maturation stage ameloblasts, located in association with intracellular vesicles and the apical membrane, and both are genetically linked to amelogenesis imperfecta [57]. Since WDR72 is a WD40-repeat containing protein, there may be a parallel to the physical interaction between NCKX2 and KIF21A [46], which is known to be mediated via the WD40-domains in KIF21A and the cytoplasmic loop of NCKX2. Such speculations await experimental investigation.

## 7. Concluding Remarks

In this review, we have focused on a variety of modalities by which K^+^-dependent Na^+^/Ca^2+^-exchangers can be regulated, including changes in cellular location, protein expression, genetics, substrate concentration, and finally, allostery. While these studies have great potential to unlock the relation between NCKX proteins, Ca^2+^ homeostasis, and physiological function, this area remains under-explored. The topic of allosteric regulation, in particular, has much promise for the future. New post-translational modifications, novel interaction partners, and a deeper mechanistic understanding of how such interactions lead to changes in NCKX transporter function are eagerly anticipated. This new knowledge will deepen our understanding regarding the control of Ca^2+^ signaling, will have important implications for transporter function in general, and has the potential to provide new therapies to treat pathological states where Ca^2+^ homeostasis is dysregulated.
